# *Arabidopsis* QWRF1 and QWRF2 Redundantly Modulate Cortical Microtubule Arrangement in Floral Organ Growth and Fertility

**DOI:** 10.3389/fcell.2021.634218

**Published:** 2021-02-09

**Authors:** Huifang Ma, Liyuan Xu, Ying Fu, Lei Zhu

**Affiliations:** State Key Laboratory of Plant Physiology and Biochemistry, College of Biological Sciences, China Agricultural University, Beijing, China

**Keywords:** floral organ development, microtubule associated protein, QWRF1, QWRF2, fertility

## Abstract

Floral organ development is fundamental to sexual reproduction in angiosperms. Many key floral regulators (most of which are transcription factors) have been identified and shown to modulate floral meristem determinacy and floral organ identity, but not much is known about the regulation of floral organ growth, which is a critical process by which organs to achieve appropriate morphologies and fulfill their functions. Spatial and temporal control of anisotropic cell expansion following initial cell proliferation is important for organ growth. Cortical microtubules are well known to have important roles in plant cell polar growth/expansion and have been reported to guide the growth and shape of sepals and petals. In this study, we identified two homolog proteins, QWRF1 and QWRF2, which are essential for floral organ growth and plant fertility. We found severely deformed morphologies and symmetries of various floral organs as well as a significant reduction in the seed setting rate in the *qwrf1qwrf2* double mutant, although few flower development defects were seen in *qwrf1* or *qwrf2* single mutants. QWRF1 and QWRF2 display similar expression patterns and are both localized to microtubules *in vitro* and *in vivo*. Furthermore, we found altered cortical microtubule organization and arrangements in *qwrf1qwrf2* cells, consistent with abnormal cell expansion in different floral organs, which eventually led to poor fertility. Our results suggest that QWRF1 and QWRF2 are likely microtubule-associated proteins with functional redundancy in fertility and floral organ development, which probably exert their effects *via* regulation of cortical microtubules and anisotropic cell expansion.

## Introduction

Flower development is essential for sexual reproduction in flowering plants. Over the past three decades, complex gene regulatory networks have been shown to control the emergence of floral primordia and the formation of different types of floral organs in a stereotypical pattern (Denay et al., 2017). A classic “ABC” model in floral organ identity specification has been raised (Bowman et al., 1991, 2012; Coen and Meyerowitz, 1991). Specification of floral organs (sepals, petals, stamens, and carpels) requires the combined activities of floral organ identity genes encoding MADS-domain transcription factors (Theißen et al., 2016). Following initiation, symmetrically arranged floral organs grow to their final shape and size; this is important for their reproductive function and for plant fertility. However, hormone deficiency, unfavorable environmental conditions, or genetic mutations leading to abnormal floral organ morphologies may eventually cause plant sterility (Reeves et al., 2012; Smith and Zhao, 2016).

Growth of floral organs relies on coordinated cell proliferation and expansion (Irish, 2010; Powell and Lenhard, 2012; Thomson and Wellmer, 2019). Transcription factors AINTEGUMENTA (ANT), JAGGED (JAG) and NUBBIN (NUB), cytochrome P450 KLUH, and E3 ubiquitin ligase BIG BROTHER (BB) have been reported to regulate cell proliferation in floral organs (Krizek, 1999; Zondlo and Irish, 1999; Krizek et al., 2000; Dinneny et al., 2004, 2006; Ohno et al., 2004; Disch et al., 2006; Anastasiou et al., 2007). However, the regulatory mechanism underlying cell expansion in the later phase of floral organ growth is largely unknown.

Cortical microtubules guide the orientation of cellulose microfibrils in the cell wall (Paredez et al., 2006; Gutierrez et al., 2009). Recently, Hervieux et al. (2016) reported that microtubules function as both stress sensors and growth regulators in *Arabidopsis thaliana*, *via* a mechanical feedback loop that regulates the growth and shape of the sepal. Signaling by rho GTPases of plants was also found to influence petal morphology in *Arabidopsis* by modulating cortical microtubules in both abaxial and adaxial epidermal cells of petals (Ren et al., 2016, 2017). Moreover, microtubule-associated proteins (MAPs) KATANIN 1 (KTN1) and INCREASED PETAL GROWTH ANISOTROPY 1 (IPGA1) were found to regulate microtubule organization, with important roles in cell expansion and petal shape (Ren et al., 2017; Yang et al., 2019a). Nevertheless, characterization of new regulators and their functions is needed to further understand the regulation of floral organ growth and flower development.

*Arabidopsis* QWRF family proteins share a highly conserved QWRF amino acid sequence and a DUF566 domain of unknown function (Pignocchi et al., 2009; Albrecht et al., 2010). One member of this family, ENDOSPERMDEFECTIVE1 (EDE1, also named QWRF5), has been shown to be an essential MAP for endosperm development (Pignocchi et al., 2009). QWRF1 (also named SNOWY COTYLEDON3, SCO3) is a peroxisome-associated protein required for plastid development. Its localization to the periphery of peroxisomes is dependent on microtubules (Albrecht et al., 2010). So far, there have been no reports about the function of QWRF2 in *Arabidopsis*.

In this study, we identified overlapping expression patterns of *QWRF1* and *QWRF2* in flowers. Severe fertility defects in the *qwrf1qwrf2* double mutant were attributed to abnormal development of floral organs. Further experiments demonstrated that both QWRF1 and QWRF2 are likely MAPs that are involved in the organization of cortical microtubule arrays, with essential roles in cell expansion, and that this regulatory mechanism is generally adopted for growth control in different floral organs.

## Materials and Methods

### Plant Materials and Growth Conditions

*Arabidopsis thaliana* ecotype Col-0 was the background for all wild-type and mutant materials in this study. Seedlings were grown on half-strength Murashige and Skoog medium with 1% sucrose in a growth chamber before transfer to soil. Seedlings/plants were grown at 22°C with a photoperiod of 16 h light/8 h dark.

T-DNA insertion lines *qwrf1-1* (SALK_072931), *sco3-3* (SALK_089815), and *qwrf2-1* (SALK_119512) were obtained from the Arabidopsis Biological Resource Center. The insertion sites of *qwrf1-1* mutant and *sco3-3* mutants were 995 bp and 1,176 bp after the start codon, respectively, and the insertion site of *qwrf2-1* mutant was 1,325 bp after the start codon. Polymerase chain reaction (PCR)-based genotyping was performed using the primers listed in Supplementary Table 1.

### Reverse-Transcription Quantitative PCR (RT-qPCR) Analysis

To quantify *QWRF1* and *QWRF2* transcripts in *qwrf1* and *qwrf2* mutants, total RNA was extracted from inflorescences and flowers using an RNA extraction kit (DP432, Tiangen, China) and reverse-transcribed with SuperScript^TM^ III (18080044, Thermo Scientific, United States). The primer pairs are listed in Supplementary Table 1. SYBR Premix Ex Taq (DRR081A, Takara Bio, Japan) was used for amplification.

### CRISPR/Cas9 Method

The target sequence of *QWRF2* was selected by the CRISPR-P (Lei et al., 2014) technique. Guide RNAs were cloned from pCBC-DT1T2 and transformed into Col as previously described (Li et al., 2020). Briefly, we designed primers with two specific sites from target gene and pCBC-DT1T2 was used as PCR template. The PCR product was cloned into pHEE401 and transformed into Col-0 using the Agrobacterium-mediated flower-dipping method (Clough and Bent, 1998). We obtained a line with a 257-bp deletion in the first exon of *QWRF2* and named it *qwrf2cas9*. The CRISPR/Cas9 constructs were then removed to ensure genetic stability. Primers are listed in Supplementary Table 1.

### Generation of Constructs and Transgenic Plants

A 2-kb region of the *QWRF1* and a 3-kb region of the *QWRF2* promoter were amplified from wild-type genomic DNA using the primers listed in Supplementary Table 1. The products were cloned into pCAMBIA1300 vectors (Cambia, Canberra, Australia), and *QWRF1/QWRF2* and GFP fusion sequences were inserted into the resulting pCAMBIA-*QWRF1pro* and pCAMBIA-*QWRF2pro* vectors, respectively, using a Clone Express II One Step cloning kit (C112-02, Vazyme, China). Sequence-verified constructs were transformed into wild-type plants by the Agrobacterium-mediated flower dipping method (Clough and Bent, 1998).

### GUS Staining and *in situ* Hybridization

For GUS staining, native promoters of *QWRF1* (*QWRF1pro*, 2057 bp fragment upstream of the start codon of *QWRF1*) and *QWRF2* (*QWRF2pro*, 3061 bp upstream of *QWRF2*) were inserted into the pCAMBIA1391 vector to drive the *GUS* reporter gene. GUS analysis was performed as previously described (He et al., 2018). Briefly, inflorescences were stained within solution containing 5-bromo-4-chloro-3-indolyl-b-D-glucuionode (X-Gluc) for 10 h at 37°C in the dark, and then destained in 70% ethanol and 30% ethanoic acid. Images were captured with an Olympus SZX16 microscope equipped with a color CCD camera (Olympus DP70) and ImagePro software (Media Cybernetics).

For *in situ* hybridization, primers (Supplementary Table 1) targeting the unique regions of *QWRF1* and *QWRF2* were used for PCR amplification to synthesize the sense and antisense probes using SP6 and T7 polymerases, respectively. Each PCR product was used as a template for *in vitro* transcription as described in the manufacturer’s protocol (11175025910, Roche, Germany). *Arabidopsis* flowers were fixed in 3.7% formol-acetic-alcohol (FAA), and *in situ* hybridization was performed as described previously (Zhang et al., 2013). A DIG Nucleic Acid Detection Kit (Roche) was used to detect the hybridized probe, and images were captured with an Olympus BX51 digital camera equipped with a Cool SNAP HQ CCD camera (Photometrics), and MetaMorph software (Universal Imaging) was used for imaging analysis.

### Agroinfiltration-Mediated Transient Expression

To generate the *35S:GFP-QWRF1* and *35S:GFP-QWRF2* constructs, we first cloned the coding sequences of *QWRF1* and *QWRF2* into the pDONR201 vector using Gateway BP Clonase II enzyme mix (11789020, Thermo Scientific), and subsequently cloned them into the pGWB506 vectors using Gateway LR Clonase enzyme mix (11791019, Thermo Scientific). *QWRF1-GFP* and *QWRF2-GFP* driven by the *pSUPER* promoter were cloned into transformed pCAMBIA1300. The resulting constructs were introduced into BY-2 tobacco (*Nicotiana tabacum*) suspension cells by a previously described *Agrobacterium* cocultivation method (An, 1985). Images were acquired with a Zeiss LSM 710 confocal microscope with a × 40 oil objective (1.3 NA).

### Protein Expression and Microtubule-Binding Assays

To obtain QWRF1 and QWRF2 proteins, *QWRF1* and *QWRF2* cDNA were transferred from pDONR207 into pET30a (+) (Novagen) and used for *in vitro* translation with a T_N_T^®^T7 Quick Coupled Transcription/Translation System (L1170, Promega, United States). The resulting proteins were incubated with pre-polymerized microtubules, centrifuged at 100,000 × *g* for 30 min at 25°C, and then analyzed by 10% SDS-PAGE (Wang et al., 2007). The Transcend Chemiluminescent Non-Radioactive Translation Detection System (L5080, Promega) was used to detect biotin-labeled QWRF1 and QWRF2 proteins.

### Light Microscopy and Scanning Electron Microscopy

To analyze fertilization rate, unfertilized ovules were counted in mature siliques to identify seed set frequency. Opened siliques were observed under an Olympus SZX16 microscope.

The flower stages were defined as reported by Smyth et al. (1990). Images of petals, sepals, stamen filaments, and stigma of stage 14 flowers from the wild type and *qwrf1qwrf2* double mutant were captured using a SZX16 microscope (Olympus). The lengths and width of petals, sepals, filaments, and stigma were measured using ImageJ software (National Institutes of Health^1^).

Clearing of stigma was performed as previously reported (Takeda et al., 2018). Briefly, inflorescences were fixed in 3.7% FAA, followed by dehydration through an ethanol series and cleared overnight in clearing solution (40 g chloral hydrate, 10 ml glycerol and 5 ml distilled water). Images were captured using an Olympus BX51 digital camera. All experiments were performed in triplicate, with 6–8 flowers measured in each experiment.

Cross-sections were cut to 2 μm thickness and stained with 0.1% (w/v) toluidine blue O in 0.1 M phosphate buffer, pH 7.0 (Ito et al., 2007). Images were captured using an Olympus BX51 digital camera.

Pollen grains on stigma were stained with aniline blue and then counted as described previously (Doucet et al., 2019). Samples were observed using an Olympus BX51 digital camera.

For staining of petal epidermal cells, stage 14 flowers were incubated in 50 μg/mL PI (propidium iodide, P4170, Sigma-Aldrich, United States) in half-strength MS liquid medium for 1 h, then observed under a Zeiss LSM 710 confocal microscope with a × 40 oil objective (1.3 NA).

The confocal analysis of ovules was performed as described previously (Cui et al., 2015). The pistils were fixed in 4% glutaraldehyde (12.5 mM cacodylate, pH6.9) and then dehydrated with ethanol gradient, clarified in benzyl benzoate: benzyl alcohol [2: 1(v/v)] overnight. Images were observed using a Zeiss LSM 710 microscope with a × 40 oil objective (1.3 NA).

Fresh material (stigma, anthers, or mature pollen grains) was spread onto the surface of adhesive tapes and observed using a scanning electron microscope (TM3000, Hitachi) at an accelerating voltage of 15 kV.

Cells expressing *35S:GFP-TUA6 (TUBULIN ALPHA-6*; Ueda et al., 1999) or *UBQ10:mCherry-MBD* (*microtubule binding domain*) were observed under a Zeiss LSM 710 confocal microscope with × 40 and × 60 oil objective (1.3 NA). Microtubule alignment was measured using fibriltool, an ImageJ plug-in, to calculate the anisotropy of the fibers (Boudaoud et al., 2014); a value close to 1 indicated strong anisotropy of the microtubules. Microtubule bundling was quantified as previously described (Higaki et al., 2010; Zhu et al., 2016). Samples were imaged with a Zeiss LSM 710 confocal laser scanning microscope. Z stacks of optical sections were taken and projected using ZEN 2012 software. Images were skeletonized and masked by manually segmenting the cell region images with ImageJ. The intensity distribution of the microtubule pixels was determined using Skewness, an ImageJ plug-in, and used as an indicator of microtubule bundling. At least 100 cells were measured.

Cells were treated with a microtubule-specific depolymerizing drug, oryzalin (36182, Sigma-Aldrich), and an actin polymerization inhibitor, Lat B (latrunculin B, L5288, Sigma-Aldrich), as previously described (Kang et al., 2017). Cortical microtubule numbers in petal abaxial epidermal cells were quantified using ImageJ as previously reported (Liu et al., 2013; Sun et al., 2015). Briefly, a vertical line was drawn perpendicularly to the majority of the cortical microtubules, and the number of cortical microtubules across the line was counted manually as the density.

## Results

### QWRF1 and QWRF2 Function Redundantly in Plant Fertility

**FIGURE 1:**
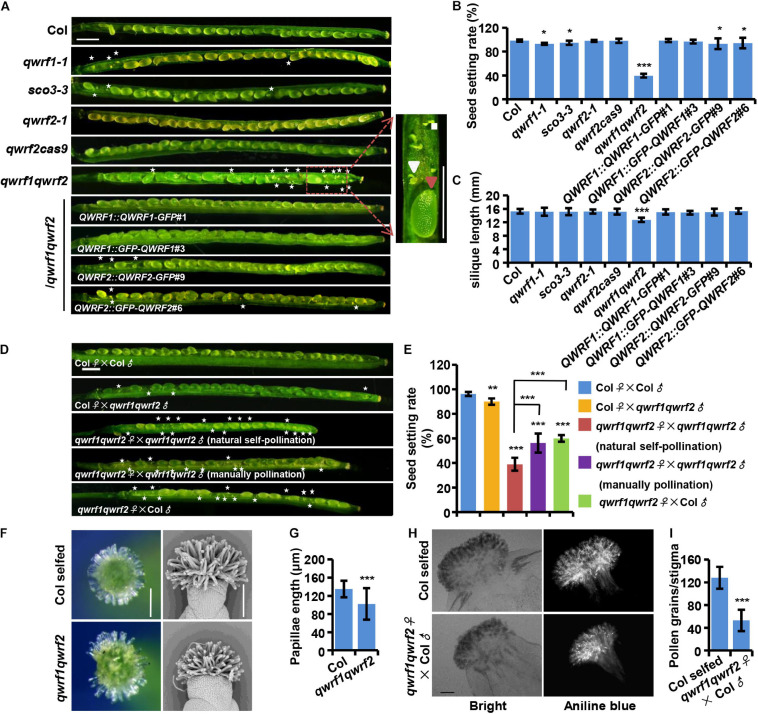
QWRF1 and QWRF2 have functionally redundant roles infertility. **(A)** Developing seeds on opened siliques, more unfertilized ovules were seen in *qwrf1* (*qwrf1-1* and *sco3-3*) single mutant and *qwrf1qwrf2* double mutant than in wild type. The siliques were shorter in *qwrf1qwrf2* compared to that in the wild type. There was no obvious difference between wild type and *qwrf2* (*qwrf2-1* and *qwrf2cass9*) single mutant. The defects in *qwrf1qwrf2* were rescued by the *qwrf1qwrf2* complementation lines (*QWRF1* or *QWRF2* cDNA constructs fused with a C-terminal GFP or N-terminal GFP). Asterisks indicate the unfertilized ovules. The close-up views shows the fertilized ovule (big and green, red arrowhead) and unfertilized ovule (small and white, white arrowhead) besides the panels. Scale bar, 1 mm. **(B)** and **(C)** Quantitative analysis of seed setting rate **(B)** and silique length **(C)** shown in panel **(A)**. The values are the mean ± SD of three independent experiments, each with at least nine siliques from three plants. *P < 0.05, ***P < 0.001, Student’s *t* test. **(D)** Fourteen days after pollination (DAP) siliques were derived from self-pollination or reciprocal crosses between the wild type and the *qwrf1qwrf2* double mutant plants. Compared with wild-type self-pollination siliques, unfertilized ovules were obviously existed no matter the *qwrf1qwrf2* was used as a male for pollen donors or as a female pollinated by the wild-type or the *qwrf1qwrf2* pollens. Manually pollination of *qwrf1qwrf2* plant can partially rescue the semi-sterile phenotype of *qwrf1qwrf2* when natural self-pollination. Asterisks indicate the unfertilized ovules. Scale bar, 1 mm. **(E)** Quantification of seed setting rate in panel **(D)**. The values are the mean ± SD of three independent experiments, each with at least nine siliques from three plants. **P < 0.01, ***P < 0.001. **(F)** Compared to wild type, the *qwrf1qwrf2* stigmas papilla cells at stage 14 appeared shorter and more centralized when observed by stereoscope (left) and scanning electron microscopy (SEM, right). Scale bar, 200 μm. **(G)** Quantification of papillae length in panel **(F)**. Values are mean ± SD of 120 cells from 10 stigmas, ***P < 0.001, Student’s *t* test. **(H)** Pollinated with wild-type pollens, much less pollen grains adhered on the *qwrf1qwrf2* stigma than on wild-type stigma. Pistils were collected at 2 h after-pollination (HAP) and pollen grains which adhered to the stigmatic papillae and stained by aniline blue were shown in the bright-field and fluorescent images, respectively. Scale bar, 100 μm. **(I)** Quantitative analysis of the adhered pollen grains numbers to each stigma from panel **(H)**. Values are mean ± SD of three independent experiments, each with 10 stigmas, ***P < 0.001, Student’s *t* test.

To better understand the regulation of plant fertility and the role of modulating microtubules in this process, we searched for lower fertility phenotypes in mutants harboring a transfer (T)-DNA insertion in previously reported genes expressed in flowers, which are likely to encode microtubule-associated proteins (Pignocchi et al., 2009; Albrecht et al., 2010). We identified a mutant line (SALK_072931) with a mild seed setting rate phenotype (Figure 1A). This mutant harbored a T-DNA insertion in the first exon of the *AT3G19570.2* gene (Supplementary Figure 1A), which encodes a member of the QWRF protein family, QWRF1 (also named SCO3, Albrecht et al., 2010). RT-PCR analysis demonstrated that it was a null mutant (Supplementary Figure 1B), and we named it *qwrf1-1*. Fourteen days after pollination (DAP), a few unoccupied spaces containing small and white ovules that were probably unfertilized (Chen et al., 2014) could be seen in *qwrf1-1* siliques. This phenomenon was rarely found in wild-type siliques at this stage. In mature *qwrf1-1* siliques, about 7.1% of seeds were aborted, significantly different from the number in the wild type (1.6%) (Figure 1B), but the mean length of siliques was similar between the *qwrf1-1* mutant (15.1 ± 1.2 mm) and the wild type (15.3 ± 0.7 mm) (Figure 1C). Similar phenotypes were observed in *sco3-3* (Figures 1A,B), a previously reported *qwrf1* knockout line (Albrecht et al., 2010).

As the phenotypes of *qwrf1-1* mutants were relatively weak, we suspected a functional overlap among QWRF proteins. *QWRF2* (*AT1G49890*) is the closest homolog of *QWRF1* in *Arabidopsis* (Pignocchi et al., 2009). Therefore, we obtained a knockout T-DNA insertion line of *QWRF2* (named *qwrf2-1*, SALK_119512) from ABRC and generated another loss-of-function allele by CRISPR/Cas9 (named *qwrf2cas9*), which had a 257-nucleotide deletion after the 352th base pair, resulting in early termination of QWRF2 protein translation (Supplementary Figure 1C). There was no significant difference in seed setting rate or silique length between the wild-type and *qwrf2* mutant lines (Figures 1B,C). We then generated a *qwrf1qwrf2* double mutant by crossing *qwrf1-1* with *qwrf2-1* and analyzed the phenotypes (Supplementary Figure 1B). Unfertilized ovules were dramatically enhanced in the double mutant at 14 DAP, and the rate of seed setting was only 40% in the *qwrf1qwrf2* mutant (Figures 1A,B). The mean length of *qwrf1qwrf2* mature siliques was significantly shorter than that in the wild type (Figure 1C). We then introduced GFP-fused QWRF1 or QWRF2, driven by the respective native promoter, into the *qwrf1qwrf2* mutant (Supplementary Figures 1D–G). Expression of either one could rescue the seed setting rate and silique length phenotypes of the double mutant (Figures 1A–C). These results confirmed that the fertility defects in the double mutant could be attributed to the simultaneous loss of function of *QWRF1* and *QWRF2*, indicating their functional redundancy. Moreover, fusion with GFP (in the N- or the C-terminus) did not interfere with the proper function of QWRF1 or QWRF2 (Figures 1A–C).

### QWRF1 and QWRF2 Have Important Roles in Floral Organ Growth

To understand how QWRF1 and QWRF2 influenced plant fertility, we first conducted reciprocal crosses between double mutant and wild-type plants. Pollination of wild-type stigma with *qwrf1qwrf2* pollens led to a mild but significant reduction in seed setting rate compared with self-pollinated wild-type plants (Figure 1D), indicating a defect in pollen development in the double mutant. Indeed, in stage 14 flowers, many *qwrf1qwrf2* mature anthers had far fewer pollen grains than wild-type anthers, and nearly 20% of *qwrf1qwrf2* pollen grains were aborted (Supplementary Figure 2). Moreover, pollinating *qwrf1qwrf2* plants with wild-type pollens caused a dramatic reduction in seed setting rate compared with either wild type self-pollinated or mutant pollen-pollinated wild-type plants (Figures 1D,E), indicating that defects in pistils contributed primarily to the fertility phenotypes of *qwrf1qwrf2* double mutants. We further analyzed the related developmental defects in pistils. Although we observed normal embryo sacs in unfertilized *qwrf1qwrf2* ovules (Supplementary Figure 3), we found abnormal stigma in the mutant: the *qwrf1qwrf2* papilla cells appeared shorter and more centralized compared with those of the wild type (Figures 1F,G). Moreover, when we used wild-type pollens to pollinate, much less pollen grain adhered on the mutant stigma than on wild-type stigma (Figures 1H,I), suggesting that the defect in papilla cells might perturb the adhesion of pollen grains on the stigma and subsequent fertilization. Furthermore, manual pollination of a *qwrf1qwrf2* plant with its own pollen grains resulted in significantly higher seed-setting rates compared with natural self-pollination (Figures 1D,E), suggesting physical barriers to self-pollination in the double mutant.

**FIGURE 2:**
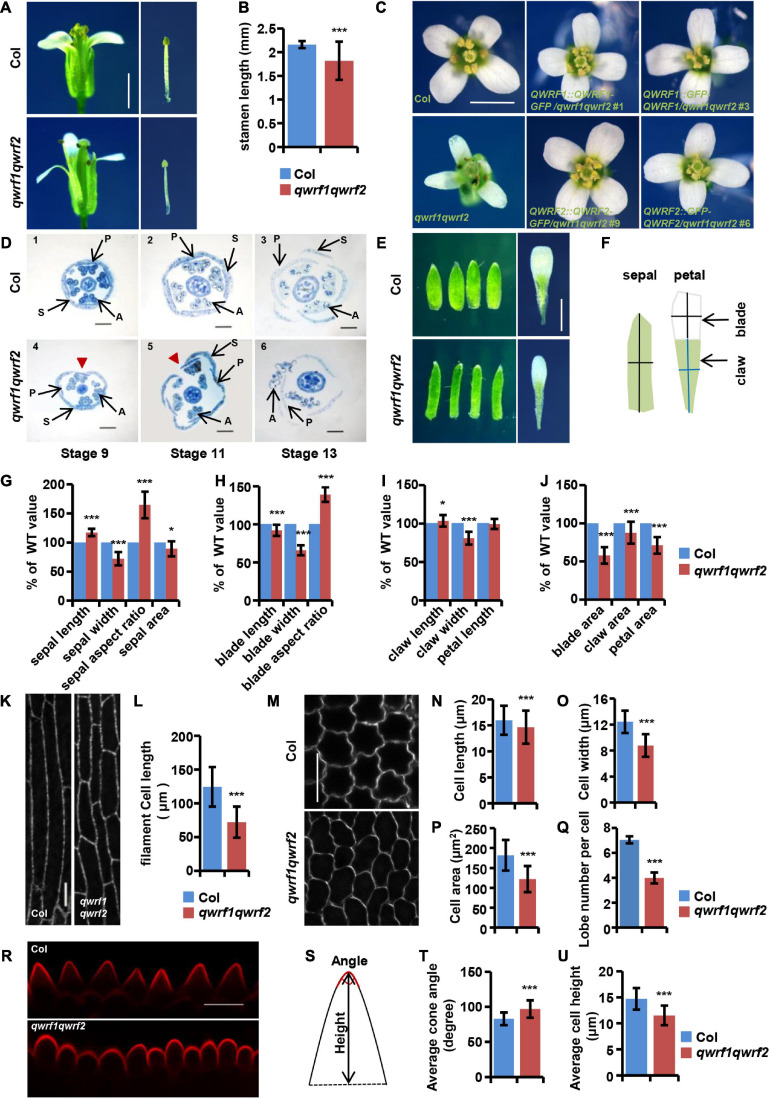
The *qwrf1qwrf2* mutant displays severe developmental defects in floral organs. **(A)** Representative dissected flowers and stamens of wild type and *qwrf1qwrf2* at stage 14. The filament length of *qwrf1qwrf2* reduced than that of wild type. Scale bar, 1 mm. **(B)** Statistics of filament length in panel **(A)**. The values are the mean ± SD three independent assays, *n* = 12. ***P < 0.001, Student’s *t* test. **(C)** Representative opened flowers of wild type, *qwrf1qwrf2* and various *qwrf1qwrf2* complementation lines. Compared with the wild-type cross-symmetrical floral organs, the floral organ morphology of the *qwrf1qwrf2* mutant was asymmetry clearly, which can be rescued by *qwrf1qwrf2* complementation lines. Scale bar, 1 mm. **(D)** Resin-embedded cross-sections of wild type (1–3) and *qwrf1qwrf2* mutant (4–6) flowers at different stages, flowers of *qwrf1qwrf2* show the disturbed sepals and petals organization. Red arrowheads indicate enlarged gap between adjacent sepals. P, petal; S, sepal; A, anther. Scale bar, 200 μm. **(E)** Compared to the wild type at stage 14, the sepals from *qwrf1qwrf2* were longer and narrower, and the petals were shorter and narrower, and both the sepal and petal area were reduced significantly. Scale bar, 1 mm. **(F)** Schematic diagram shows how the sepal and petal length and width were measured. **(G)** Quantification of sepal parameters in panel **(E)**. Values are mean ± SD of 20 sepals from different plants. *P < 0.05, ***P < 0.001, Student’s *t* test. **(H–J)** Quantification of petal parameters of wild type and *qwrf1qwrf2* in panel **(E)**. Values are mean ± SD of three independent assays, from at least 36 petals. *P < 0.05, ***P < 0.001, Student’s *t* test. **(K)** Epidermal cell in the middle region of stage 14 stamen filament from wild type and *qwrf1qwrf2* by transforming *UBQ10:mCherry-MBD* construct. Scale bar, 10 μm. **(L)** The stamen filament cells in wild type were longer than in *qwrf1qwrf2* mutant. Values are mean ± SD. *n* = 120 cells, ***P < 0.001, Student’s *t* test. **(M)** Cells from the blade regions of petal abaxial epidermis of wild type and *qwrf1qwrf2* mutant at stages 14 by PI staining. The *qwrf1qwrf2* petal abaxial epidermis cell shape changed obviously compared with that in wild type. Scale bar, 10 μm. **(N–R)** Quantification of cell parameters from petal abaxial epidermis cells in panel **(M)**. **(N)** Reduced cell length in *qwrf1qwrf2*. **(O)** Reduced cell width in *qwrf1qwrf2*. **(P)** Reduced cell area in *qwrf1qwrf2*. **(Q)** Reduced number of lobes per cell in *qwrf1qwrf2*. Values are mean ± SD of more than 500 cells of 6–8 petals from different plants. ***P < 0.001, Student’s *t* test. **(R)** Conical cells shape changed between wild type and *qwrf1qwrf2* mutant at stage 14 by PI staining. Scale bar, 10 μm. **(S)** The carton illustrating how the conical cell angles and heights were measured. **(T,U)** Quantitative analysis conical cell parameters from panel **(R)**. The angle of conical cell was increased **(T)** and conical cell heights decreased **(U)** in *qwrf1qwrf2* mutant than in wild type. Values are mean ± SD of more than 400 cells of 8 petals from different plants. ***P < 0.001, Student’s *t* test.

There were multiple developmental defects in *qwrf1qwrf2* flowers, including (1) shorter filaments such that the anthers hardly reached the stigma (Figures 2A,B); (2) a deformed floral organ arrangement lacking the cross-symmetry usually seen in the wild type, with bending petals sometimes forming an obstacle between anthers and stigma (Figures 2C,D); and (3) generally smaller and narrower petals and sepals compared with the wild type (Figures 2E–J). All these phenotypes were complemented by expression of GFP-fused QWRF1 or QWRF2 in *qwrf1qwrf2* mutant (Figure 2C).

Using RT-RCR we found that both *QWRF1* and *QWRF2* were constitutively expressed in plants, with high levels in flowers (Supplementary Figure 4A). The expression of *QWRF1* and *QWRF2* in sepals, petals, stamens, stamen filaments, and pistils was further confirmed by GUS activity assay and *in situ* hybridization analysis (Supplementary Figures 4B,C). These results were consistent with those previously reported by Albrecht et al. (2010) as well as those in the Genevestigator database^2^.

The above evidence demonstrates the important and redundant roles of QWRF1 and QWRF2 in the development of the floral organ. Loss of function of both genes led to developmental defects in flowers, including shorter stamen filaments and abnormal arrangements in floral organs, which probably caused severe physical obstacles that hindered natural pollination and reduced the subsequent seed setting rate.

### QWRF1 and QWRF2 Are Involved in Anisotropic Cell Expansion

In plants, growth of organs to their final size and shape depends on cell proliferation followed by cell expansion (Powell and Lenhard, 2012). Phenotypes such as shorter stamen filaments, and narrower and smaller petals and sepals in *qwrf1qwrf2* flowers suggest possible defects in polar cell expansion. To confirm this hypothesis, we analyzed cell morphology in various floral tissues. Besides shorter papilla cells (Figures 1F,G), the epidermal cells of the stamen filament were significantly shorter than those in the wild type (Figures 2K,L). Moreover, we observed adaxial and abaxial epidermal cells of petal blades from stage 14 flowers by PI staining. As shown in Figures 2M–P, *qwrf1qwrf2* abaxial petal epidermal cells had decreased average cell length, width, area, and reduced lobe numbers (Figure 2Q) compared with the wild type, indicating a reduction in cell expansion.

We also observed alterations of the shapes of conical cells in petal adaxial epidermis (using a method reported by Ren et al., 2017; Figure 2R). Quantitative analyses revealed a larger-than-wild-type cone angle in *qwrf1qwrf2* conical cells (Figures 2S,T), which lacked the pointed apex usually seen in the wild type, and a decrease in the average cell height (Figure 2U). These results suggest that QWRF1 and QWRF2 have a general role in the regulation of anisotropic cell expansion during floral organ growth.

### QWRF1 and QWRF2 Associate With Microtubules *in vitro* and *in vivo*

**FIGURE 3:**
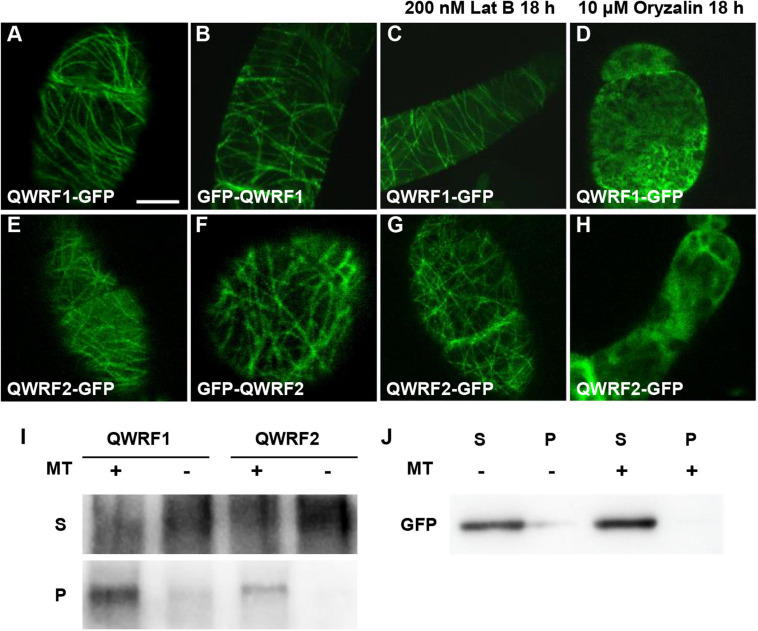
QWRF1 and QWRF2 are associating with microtubule *in vitro* and *in vivo*. **(A–D)** Subcellular localization of QWRF1. **(E–H)** Subcellular localization of QWRF2. Confocal microscopy images of the tobacco BY-2 suspension cells transiently expressing *pSUPER:QWRF1-GFP* **(A,C,D)**, *35S:GFP-QWRF1* **(B)**, *pSUPER:QWRF2-GFP* **(E,G,H)** and *35S:GFP-QWRF2* **(F)**. All these construction exhibited filamentous structures in tobacco BY-2 suspension cells, when treated with various drugs for 18 h, filamentous structures visualized in this cell remained intact in the presence of 200 nM Lat B (actin polymerization inhibitor) treatment **(C,G)**, but these structures were disrupted by 10 μM oryzalin (microtubule-specific depolymerized drug) treatment **(D,H)**. Scale bar, 10 μm. **(I)** *In vitro*-biotinylated-lysine-labeled QWRF1 or QWRF2 protein expressed in a cell-free system was co-sedimented with (+) or without (−) taxol-stabilized microtubules. After high-speed centrifugation, QWRF1 and QWRF2 proteins could be detected in pellets with microtubules. **(J)** GFP was used as a negative control, which showed no preferential co-sedermentated with microtubules. MT, microtubules; S, supernatants; P, pellets.

To better understand the function of QWRF1 and QWRF2, we investigated the subcellular localization pattern of these two proteins. As barely any fluorescence was detected in complementary lines expressing GFP-fused QWRF1 or QWRF2 driven by their native promoter, we used the *pSUPER* promoter to drive GFP-fused QWRF proteins and transiently expressed them in tobacco BY-2 suspension cells. Regardless of which terminus was fused with GFP, QWRF1 were localized to a filament-like structure that could be disrupted by microtubule-disrupting drug oryzalin but not by microfilament-disrupting drug Lat B (Figures 3A–D). This suggested that QWRF1 co-localized with microtubules in BY-2 cells. QWRF2 showed a similar localization pattern (Figures 3E–H). To further verify whether QWRF1 and QWRF2 were MAPs, we performed an *in vitro* co-sedimentation assay. Owing to the difficulty of obtaining purified recombinant QWRF1 and QWRF2 proteins using a prokaryotic expression system, we used *in vitro* coupled transcription/translation to express QWRF proteins as previously described (Pignocchi et al., 2009). Biotinylated-lysine-labeled QWRF1 or QWRF2 protein was, respectively, incubated with or without paclitaxel-stabilized pre-polymerized microtubules before high-speed centrifugation. Both QWRF1 and QWRF2 were co-sedimented with pre-polymerized microtubules in the pellets, indicating their direct association with microtubules *in vitro* (Figures 3I–J, Supplementary Figure 5). These *in vivo* and *in vitro* results were consistent with our expectations, as previous studies have shown that QWRF1/SCO3 links the microtubule, and another QWRF family protein EDE1 is a MAP (Pignocchi et al., 2009; Albrecht et al., 2010).

### QWRF1 and QWRF2 Modulate Cortical Microtubule Arrangement

**FIGURE 4:**
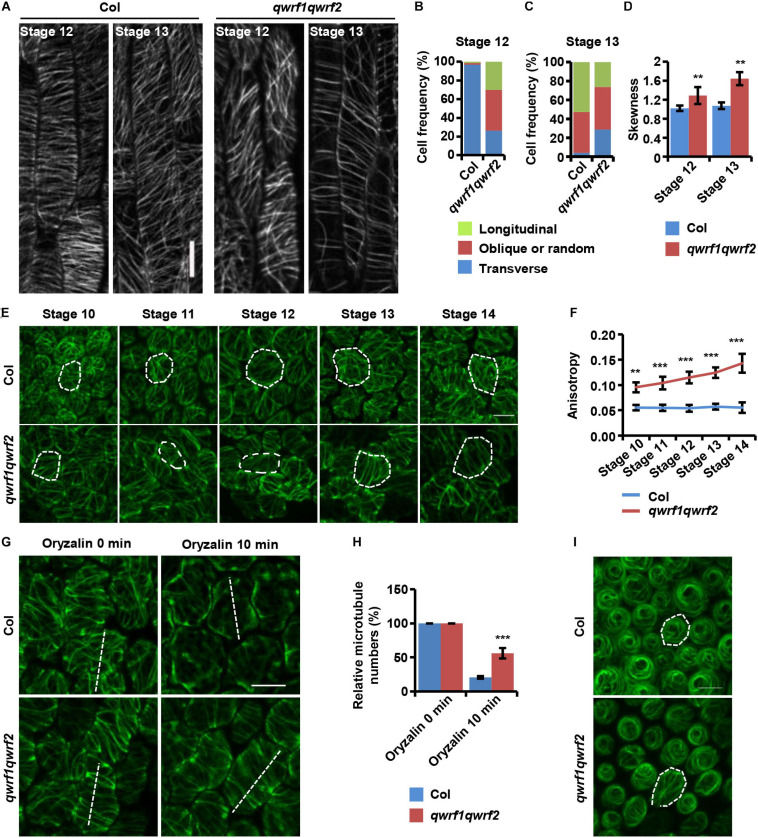
QWRF1 and QWRF2 affect cortical microtubule organization and stability in floral organ cells. **(A)** *UBQ10:mCherry*-*MBD*-labelled cortical microtubules in wild-type and *qwrf1qwrf2* stamen filament epidermal cells. The cortical microtubules array in *qwrf1qwrf2* stamen filament epidermal cells is greatly altered compared with that in wild type. Scale bar, 20 μm. **(B,C)** Frequency of microtubule orientation patterns in wild-type and *qwrf1qwrf2* upper stamen filament epidermal cell at stage 12 and 13, measured by fibriltool, an Image J plug-in as described in the method. *n* ≥ 150 cells. **(D)** Quantification of microtubule bundling (Skewness) from confocal optical images in panel **(A)**. The microtubule bundling was increased in *qwrf1qwrf2* stamen filament epidermal cells. Values are mean ± SD. *n* ≥ 100 cells, **P < 0.01, Student’s *t* test. **(E)** Cortical microtubules of abaxial epidermal cells in petal blades of wild type and *qwrf1qwrf2* with a *35S:GFP*-*TUA6* background. The microtubule arrays in *qwrf1qwrf2* petal at stage 10–14 abaxial epidermal cells were more orderly. The white dotted lines depict cell outlines. Scale bar, 10 μm. **(F)** The microtubule alignment in panel **(E)** was measured by fibriltool, an Image J plug-in as described in the method. The anisotropy close to 1 represents contained more highly ordered cortical microtubule (CMT) arrays transversely oriented relative to the axis of cell elongation. Values are mean ± SD. *n* ≥ 200 cells. ***P < 0.001, Student’s *t* test. **(G)** The organization of cortical microtubules in *qwrf1qwrf2* cells is insensitive to treatment with 20 μM oryzalin for 10 min. Scale bar, 10 μm. **(H)** Fifteen-μm of white dashed lines cross the cortical microtubules **(G)**, and the number of cortical microtubules across the line was measured as the density. Three repeated measurements were performed and at least 100 cells were used. Values are mean ± SD of more than 100 cells. ***P < 0.001, Student’s *t* test. **(I)** Cortical microtubules were observed in conical cells from opened flower petals of wild type and *qwrf1qwrf2* mutant stably expressing *35S:GFP*-*TUA6*, respectively. The white dotted lines depict cell outlines. Scale bar, 10 μm.

In plant cells, cortical microtubule arrays influence anisotropic cell expansion by guiding the deposition and orientation of cellulose microfibrils (Fujikura et al., 2014; Yang et al., 2019b). Therefore, regulation of the organization and dynamics of cortical microtubule arrays is important for the polar expansion of various cell types, and subsequently affects cell and organ morphogenesis. The above evidence showed obvious cell-expansion defects in various types of floral cells, and revealed the abnormal morphology of sepals, petals, and stamen filaments in the *qwrf1qwrf2* double mutant (Figure 2). Given that both QWRF1 and QWRF2 are suggested as MAPs, we proposed that they might exert their functions in anisotropic cell expansion and floral organ morphogenesis through modulation of cortical microtubule arrays. To test this hypothesis, we compared the cortical microtubule arrangements in epidermal cells of stamen filaments and petals between the *qwrf1qwrf2* double mutant and the wild type. As mentioned above, the *qwrf1qwrf2* mutant had shorter stamen filament epidermal cells than the wild type. To visualize the cortical microtubules in these cells, *UBQ10:mCherry-MBD* was introduced into the *qwrf1qwrf2* double mutant by crossing. As filament elongation starts at flower stage 12 and ends at stage 13 (Acosta and Przybyl, 2019), we observed stamen filaments at these two stages. At stage 12, most cortical microtubules were parallel and transversely oriented in the wild type, which is consistent with the fast cell elongation at this stage (Figure 4A). However, in *qwrf1qwrf2* cells, far fewer microtubules were transversely oriented compared with the number in wild-type cells (Figure 4B). At stage 13, when cell elongation ends, cortical microtubules were arranged obliquely in both the wild type and *qwrf1qwrf2* double mutant (Figure 4C). Moreover, compared with wild-type cells, the bundling of microtubules in *qwrf1qwrf2* cells was significantly higher according to the skewness analysis (Figure 4D).

Next, we observed cortical microtubule arrays in petal epidermal cells by stably expressing *35S* promoter-driven *GFP-TUA6* in the wild type and *qwrf1qwrf2* double mutant. As shown in Figure 2, the *qwrf1qwrf2* mutant had shorter and narrower petal blades, and consistently shorter and narrower abaxial epidermal cells. Quantitative analyses also revealed that *qwrf1qwrf2* cells had much fewer lobes than wild-type cells (Figure 2Q), indicating a stronger restriction of lateral cell expansion. Consistently, we found sparser but more orderly cortical microtubules in *qwrf1qwrf2* abaxial petal epidermal cells than in wild-type cells throughout flower stages 10–14 (Figures 4E,F). After treatment with oryzalin, there were more intact microtubule filaments in mutant cells, indicating that microtubules were more stable when both QWRF1 and QWRF2 were absent (Figures 4G,H).

Given the change in cell shape of petal adaxial conical cells in the *qwrf1qwrf2* mutant (Figure 2R), we further investigated whether QWRF1 and QWRF2 affected microtubule organization in these cells. Similar to previous reports (Ren et al., 2017), microtubule arrays in wild-type cells displayed a well-ordered circumferential orientation. However, in *qwrf1qwrf2* mutant cells, microtubule arrays were randomly oriented (Figure 4I), consistent with the mutant conical cells having larger cone angle but shorter cell height (Figures 2T,U; Ren et al., 2017).

## Discussion

Organ growth is essential for floral organs to achieve their proper morphology and fulfill their functions. Spatial and temporal control of anisotropic expansion following initial cell proliferation is important for organ growth (Irish, 2010). However, the molecular mechanism underlying the regulation of floral organ growth is largely unknown. Recently, cortical microtubules have been reported to guide the growth and shape of sepals and petals by acting as both mechanical stress sensors and growth regulators (Hervieux et al., 2016; Yang et al., 2019b). In this study, we characterized a *qwrf1qwrf2* double mutant with defects in many aspects of flower development, including abnormal size and shape of sepals and petals, short stamen filaments and papilla cells, and an altered symmetric arrangement of floral organs (Figure 2). These defects represented physical barriers to successful sexual reproduction. However, both *qwrf1* and *qwrf2* single mutants showed few defects in flower development and sexual reproduction (although *qwrf1* showed a weak reduction in seed setting rate), indicating the redundant functions of QWRF1 and QWRF2 in floral organ growth and plant fertility. Nevertheless, the floral organs of *qwrf1qwrf2* double mutant are in four whorls, suggesting that QWRF1 and QWRF2 are not critical for floral meristem establishment and organ identity.

There were significant differences in the size and shape of epidermal cells in petals and stamen filaments between the wild type and the double mutant, indicating a role for QWRF1 and QWRF2 in anisotropic cell expansion. *In vitro* and *in vivo* analyses demonstrated that QWRF1 and QWRF2 were associated with microtubules. Moreover, epidermal cells of *qwrf1qwrf2* petals and stamen filaments had cortical microtubule arrays with sparse microtubule bundles in an altered orientation compared with the wild type. Overall, we concluded that QWRF1 and QWRF2 are required for proper growth and morphology of floral organs and thus for plant fertility, and probably function *via* modulating microtubule-dependent anisotropic cell expansion during organ growth.

QWRF1/SCO3 contains a C-terminal PTS1 (peroxisomal-targeting signal type 1) domain, tripeptide SRL, which targets the periphery of peroxisomes in *Arabidopsis* cultured cells. Interestingly, GFP:SCO3ΔSRL, which lacking the peroxisome location, was unable to complement the phenotype of *sco3-1* mutant as determined by chlorophyll content in cotyledons (Albrecht et al., 2010). However, in our study, we found that expressing QWRF1ΔSRL was able to rescue floral organ growth and fertility of *qwrf1qwrf2* plants (Supplementary Figure 6), suggesting that the effects of QWRF1 on floral organ growth and fertility are unrelated to its peroxisome association. Consistently, QWRF2 has no PTS1 domain but being associated with microtubules, and being functionally redundant with QWRF1.

We also observed incomplete anther dehiscence, and shriveled and shrunken pollen grains in *qwrf1qwrf2* opening flowers; how these two proteins regulate male gametophyte development needs further study. Given that EDE1/QWRF5, another QWRF family member, colocalizes with mitotic microtubules during endosperm development (Pignocchi et al., 2009), whether QWRF1 and QWRF2 participate in microsporogenesis *via* binding to and regulating mitotic microtubules is also worthy of further investigation. Notably, the *qwrf1qwrf2* ovules had normal embryo sacs (Supplementary Figure 3), indicating that they are not involved in megasporogenesis during flower development.

## Data Availability Statement

The datasets presented in this study can be found in online repositories. The names of the repository/repositories and accession number(s) can be found in the article/Supplementary Material.

## Author Contributions

LZ, YF, and HM designed the project. HM and LX performed the experiments and analyzed the data. LZ and HM wrote the manuscript. YF revised the manuscript. All authors have contributed significantly to this work and all authors are in agreement with the contents of the manuscript.

## Conflict of Interest

The authors declare that the research was conducted in the absence of any commercial or financial relationships that could be construed as a potential conflict of interest.

## Footnotes

1 http://rsb.info.nih.gov/ij

2 www.genevestigator.ethz.ch
