# Supplementary material for: Arabidopsis QWRF1 and QWRF2 Redundantly Modulate Cortical Microtubule Arrangement in Floral Organ Growth and Fertility
Source: Front Cell Dev Biol. 2021 Feb 9;9:634218. doi: 10.3389/fcell.2021.634218 (PMC7901996; doi:10.3389/fcell.2021.634218)
Supplement: Supplementary file 1 [file Data_Sheet_1.pdf]

Supplementary Material

The following materials are available in the online version of this article.

Supplementary Figure 1. Identification of mutants and analyses of the expression of *QWRF1* and *QWRF2* in different lines.

Supplementary Figure 2. The pollen development was disturbed in the *qwrf1qwrf2* double mutant.

Supplementary Figure 3. The development of *qwrf1qwrf2* female gametophyte is normal.

Supplementary Figure 4. Similar expression pattern of *QWRF1* and *QWRF2* in floral organ.

Supplementary Figure 5. QWRF1 and QWRF2 are associating with microtubule *in vitro*.

Supplementary Figure 6. QWRF1 C-terminal PTS1 domain does not contribute to its function in flower development and fertility.

Supplementary Table 1. Primers Information.

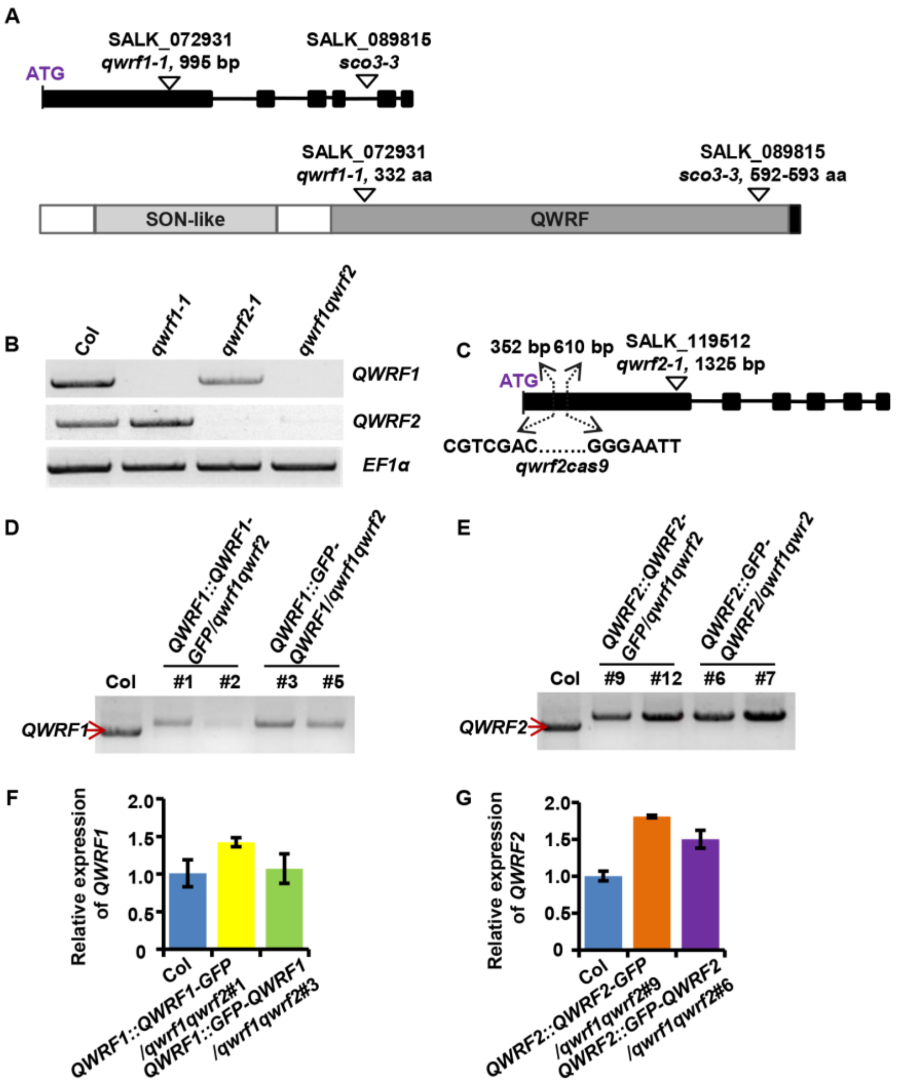

Supplementary Figure 1. Identification of mutants and analyses of the expression of *QWRF1* and *QWRF2* in different lines.

(**A**) Schematic diagrams of the *Arabidopsis thaliana QWRF1* gene and domain structure of the QWRF1 protein in T-DNA insertion mutant seedlings. Vertical line indicate ATG, blocks represent exons, and horizontal lines denote introns. The inverted triangles identified the insertion sites of T-DNA. (**B**) RT–PCR analysis of the expression of *QWRF1* and *QWRF2* in wild type, *qwrf1-1* (SALK_072931), *qwrf2-1* (SALK_119512), and *qwrf1qwrf2* double mutant. Relative amounts of gene expression were normalized to those of *EF1a*. (**C**) Schematic diagrams of the *Arabidopsis thaliana QWRF2* gene in CRISPR/Cas9 (dotted arrows) and T-DNA insertion (inverted triangle) mutant seedlings. Vertical line indicate ATG, blocks represent exons, and horizontal lines denote introns. (**D**) and (**E**) RT–PCR analysis of the expression of *QWRF1* or *QWRF2* with different GFP fusion in the *qwrf1qwrf2* complementation lines (*QWRF1* or *QWRF2* cDNA constructs fused with a C-terminal GFP or N-terminal GFP). *QWRF1* (**D**) and *QWRF2* (**E**) in wild type were uses as control. (**F**) The expression of *QWRF1* in wild type and the *qwrf1qwrf2* complementation lines. (**G**) The expression of *QWRF2* in wild type and the *qwrf1qwrf2* complementation lines. Total RNAs were extracted from seedlings of 7-d-old plants and relative transcript levels were examined by RT-qPCR analysis. Error bars indicate ± SD of three independent experiments.

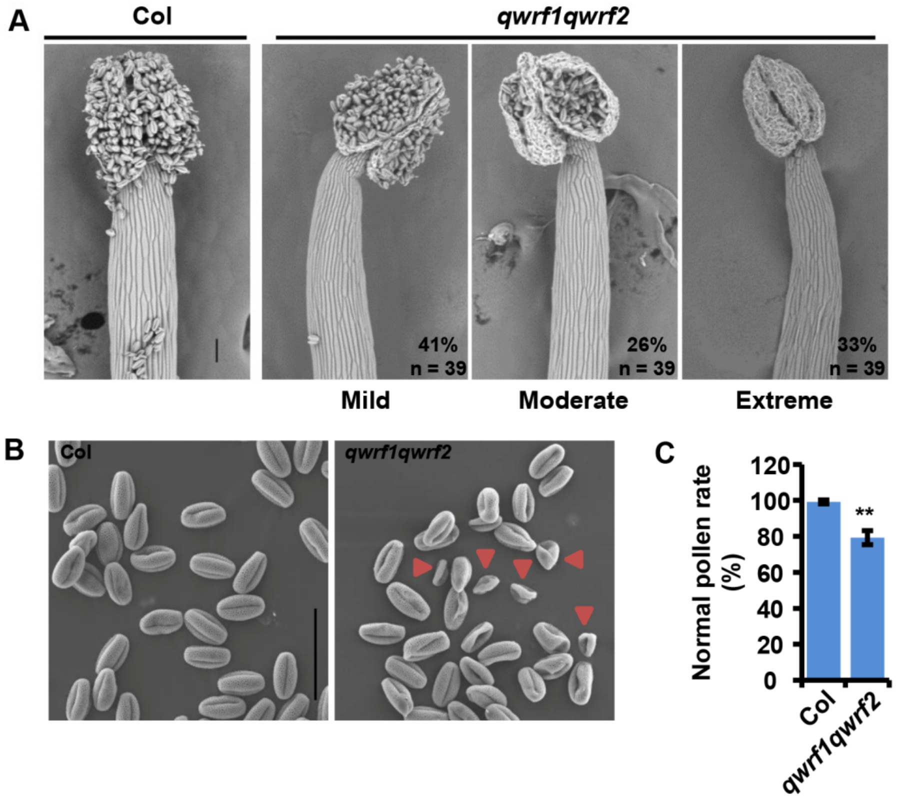

Supplementary Figure 2. The pollen development was disturbed in the *qwrf1qwrf2* double mutant.

(**A**) The anthers of stage 14 flowers were examined by SEM. Far less pollen grains were present in the *qwrf1qwrf2* mutant anthers when compared with the wild type. The *qwrf1qwrf2* anthers showed mild, moderate, and extreme phenotypes. Scale bar, 50 μm. (**B**) Dehisced pollens produced of the wild type and *qwrf1qwrf2* were viewed by SEM. Aborted pollen grains were existed in *qwrf1qwrf2* (red arrowheads). Scale bar, 10 μm. (**C**) Pollen morphology statistics. The normal or abnormal pollen grains that were derived from (**B**) were quantified. The values are the mean ± SD of three independent experiments, n = 300 pollen grains. **P < 0.01, Student's *t* test.

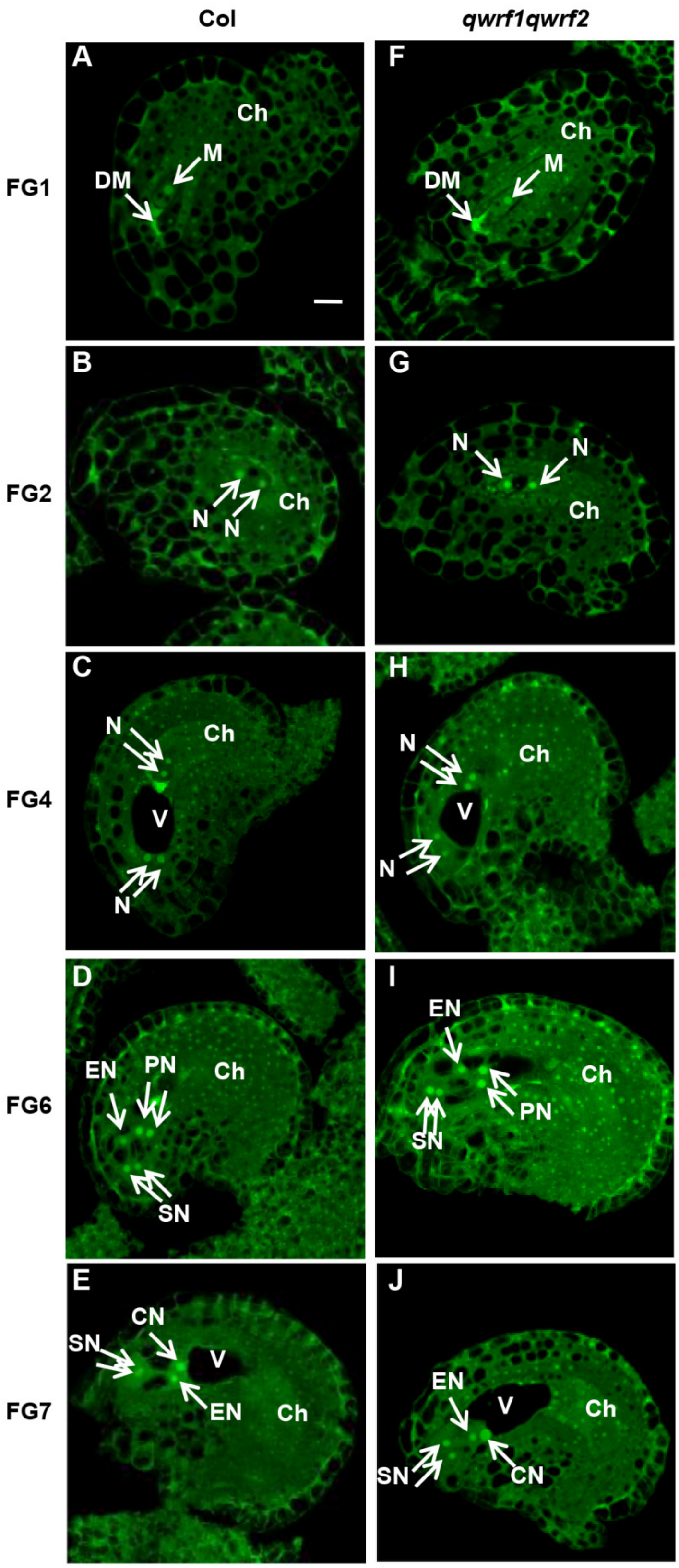

Supplementary Figure 3. The development of *qwrf1qwrf2* female gametophyte is normal.

Confocal optical section showing the development of the female gametophyte in the wild type

(**A**-**E**) and the *qwrf1qwrf2* double mutant plants (**F**-**J**). (**A**) and (**F**) The female gametophyte (FG)1: the development of embryo sac give rise to one functional megaspore and degenerating megaspores close to the micropyle; (**B**) and (**G**) FG2：the functional megaspore have a nuclear mitotic division to form a two-nucleate embryo sac; (**C**) and (**H**) FG4：the embryo sac contained four-nucleate and a large central vacuole; (**D**) and (**I**) FG6: the polar nuclei meet each other, one egg nucleus, and two synergid nuclei are visible; (**E**) and (**J**) FG7: the mature embryo sac has one central nucleus, one egg nucleus, and two synergid nuclei. Ch, chalazal end; CN, central nucleus; DM, degenerated megaspore; M, the functional megaspore; EN, egg nucleus; N, nucellus; PN, polar nuclei; SN, synergid nuclei; V, large vacuole. Scale bar, 10 μm.

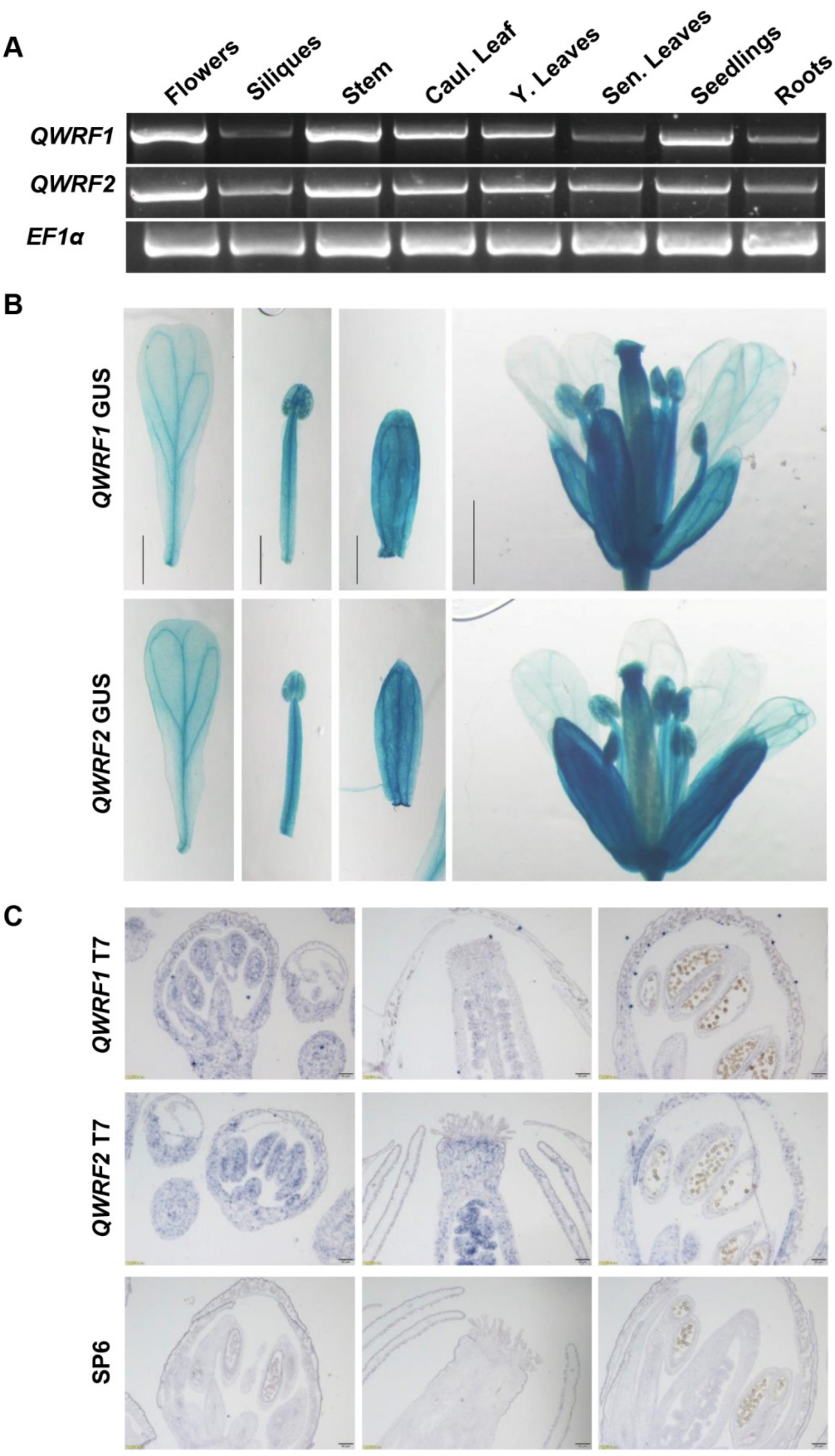

Supplementary Figure 4. Similar expression pattern of *QWRF1* and *QWRF2* in floral organ.

(**A**) RT-PCR of *QWRF1* and *QWRF2* transcripts from total RNA isolated from flowers; 2 to 4 d after pollination (DAP) siliques; stem; cauline (Caul) leaves; young (Y) leaves; senescent (Sen) leaves; 7-d-old Seedlings and 10-d-old Roots. *EF1α* was used as control. Both *QWRF1* and *QWRF2* were highly expressed in flower organs, also expressed in other tissues. There were 35

cycles used for the RT-PCR for *QWRF1* or *QWRF2*, and 28 cycles for *EF1a*. (**B**) GUS activity assay of plants transformed with *QWRF1pro-GUS* or *QWRF2pro-GUS*. Both *QWRF1* and *QWRF2* were highly expressed in the whole floral organs, including sepals, petals, stamens, and pistils. Scale bar, 1 mm. (**C**) *In situ* analysis of *QWRF1* and *QWRF2* mRNA expression in developing flower buds and pistil tissue. Longitudinal section through early inflorescence (stage 5-6, left), carpel and ovules (middle) and older inflorescence at stage 11 flower (right), revealing that *QWRF1* and *QWRF2* were expression throughout the different stage of floral organ development. Sense probe (SP6) in the same stage of flower buds was used as negative controls. Scale bar, 50 μm.

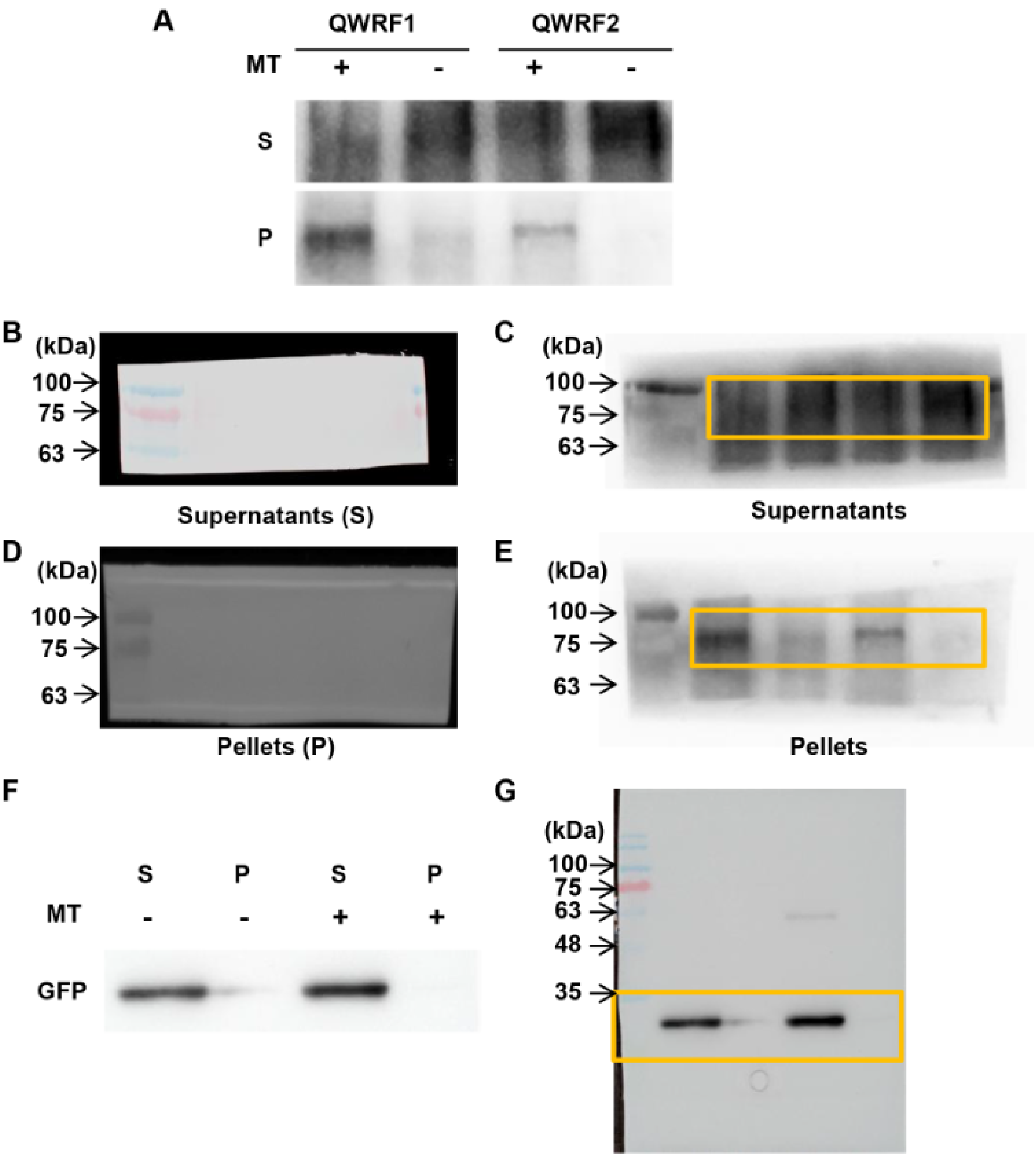

Supplementary Figure 5. QWRF1 and QWRF2 are associating with microtubule *in vitro*.

(A) *In vitro*-biotinylated-lysine-labeled QWRF1 or QWRF2 protein expressed in a cell-free system was co-sedimented with (+) or without (−) taxol-stabilized microtubules. After high-speed centrifugation, QWRF1 and QWRF2 proteins could be detected in pellets with microtubules. (A) The image was cropped from (C) and (E); the yellow box indicates the selected sections from the original blotting. Owing to the low yield of QWRF proteins from the cell-free system, in order to reduce noise interference, only the part of the SDS-PAGE gel around the expected molecular weight of the QWRF proteins was transferred to the nitrocellulose membrane (B and D). Following western blotting, the proteins were visualized by streptavidin–horseradish peroxidase followed by chemiluminescent detection (C and E). (F) GFP was used as a negative control that did not co-sediment with microtubules. (F) The image was cropped from (G). MT, microtubules; S, supernatants; P, pellets.

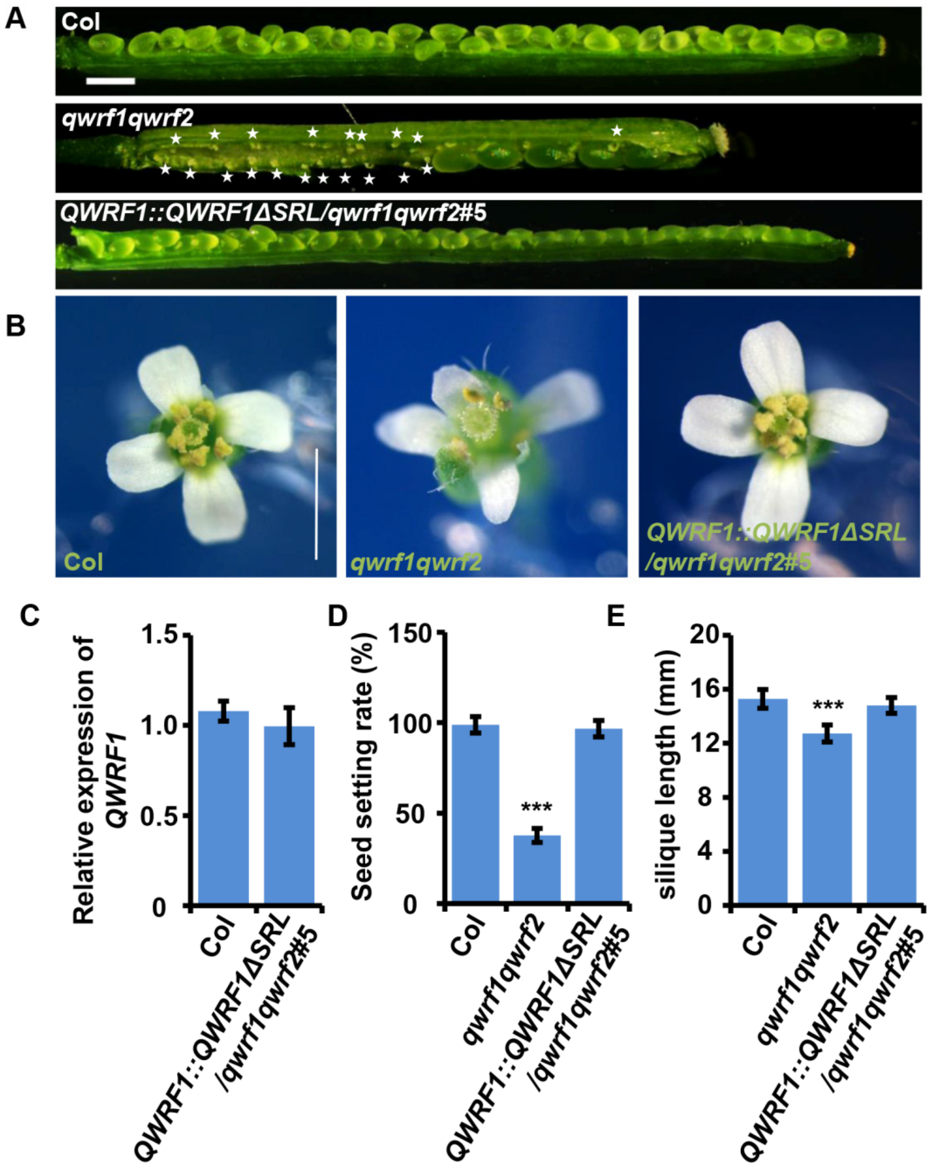

Supplementary Figure 6. QWRF1 C-terminal PTS1 domain does not contribute to its function in flower development and fertility.

(**A**) The fertility phenotype of *qwrf1qwrf2* can be rescued by QWRF1 mutant protein lacking the C-terminal PTS1 domain (QWRF1*Δ*SRL). Scale bar, 1 mm. (**B**) The defects of floral organs in *qwrf1qwrf2* can be rescued by expressing QWRF1ΔSRL. Scale bar, 1 mm. (**C**) Detection relative expression level of *QWRF1* in *QWRF1::QWRF*1*ΔSRL*/*qwrf1qwrf2* by quantitative RT-PCR. (**D**) Quantification analysis of seed setting rate in (**A**). Error bars indicate ± SD. n = 20, ***P< 0.001, by Student's *t* test. (**E**) Quantification analysis of average silique length in (**A**). Error bars indicate ± SD. n = 20, ***P< 0.001, by Student's *t* test.

Supplementary Table 1. Primers Information.

Primer name	Primer sequence
SALK_072931 LP	GAGGCTTAAGCATTTGCATTG
SALK_072931 RP	GGAGGGGAAGTTCTGAATCTG
SALK_089815 LP	GCACGTTAAGAAACATGAAAGC
SALK_089815RP	CGTGTAAACGCAGAGGAGATC
SALK_119512 LP	CGGCAAGAAGGAATCACTAAG
SALK_119512RP	GACTAATAGCCGGTTAAGGCG
QWRF2DT1-BsF	ATATATGGTCTCGATTGGACCGTCGTCGACCCTCGGGTT
QWRF2DT1-F0	TGGACCGTCGTCGACCCTCGGGTTTTAGAGCTAGAAATAGC
QWRF2DT2-R0	AACGGAGCCTCACGGAGGGGGACAATCTCTTAGTCGACTCTAC
QWQF2DT2-BsR	ATTATTGGTCTCGAAACGGAGCCTCACGGAGGGGGACAA
Cas9-U626-IDF	TGTCCCAGGATTAGAATGATTAGGC
Cas9-U629-IDF	TTAATCCAAACTACTGCAGCCTGAC
Cas9-U629-IDR	GTTGATGGATCGAAAGAAGAGGGCT
QWRF1RT-PCR-F	ATGGTGGCTGCGATTCCTCA
QWRF1RT-PCR-R	TAGCCGGCTAAGTTGTATTATGTGC
QWRF2RT-PCR-F	ATGGTTGCTGCAGCGATTTCA
QWRF2RT-PCR-R	CTACAATTGCGGAGTCAAGCT
EF1α RT-F	GCACGCTCTTCTTGCTTTCAC
EF1α RT-R	GGGCTCCTTCTCAATCTCCTTAC
QWRF1qPCR-F	GCTCGAACAGTGTCAAGGAT
QWRF1qPCR-R	AGCCGGCTAAGTTGTATTATGT
QWRF2qPCR-F	CAGCACACTTCGTCTACCAATA
QWRF2qPCR-R	ACATCGACGGCTGAACTAAC
EF1α qPCR-F	ACGCTCTTCTTGCTTTCACC
EF1α qPCR-R	GAGATTGGCACAAATGGGAT
HindIII-QWRF1pro-F	AAGCTTACAGACTAGAAAGGTATGCTCTTG
PstI-QWRF1pro-R	CTGCAG TATCTACGAGAAATGTCTCGCT
SalI-QWRF1pro-R	GTCGAC TATCTACGAGAAATGTCTCGCT

PstI-eGFP-F	CTGCAGATGGTGAGCAAGGGCGAGGAG
SalI-eGFP-R	GTCGACCTTGTACAGCTCGTCCATGCC
SalI-QWRF1-F	GTCGACATGGTGGCTGCGATTCCTCA
KpnI-QWRF1-R1	GGTACCTTATAGCCGGCTAAGTTGTATTATGTGC
KpnI-QWRF1-R2	GGTACCTAGCCGGCTAAGTTGTATTATGTGC
KpnI-QWRF1△SRL-R	GGTACCTTAAAGTTGTATTATGTGCGTCTT
EcoRI-QWRF2Pro-F	CATGATTACGAATTCCATTGTGTCTTTGAAACTAAA
KpnI-QWRF2Pro-R	CGGGTACCTCTCGTCGGAAAAAACTATTC
KpnI-QWRF2-F	CCGACGAGAGGTACCATGGTTGCTGCAGCGATTTCA
BamHI-QWRF2-F	ATGGACGAACTGTACATGGTTGCTGCAGCGATTTC
PstI-QWRF2-R1	GCTCCCGCTCCCGCTCCCCAATTGCGGAGTCAAGCTGC
PstI-QWRF2-R2	GCCAAGCTTGCATGCCTACAATTGCGGAGTCAAGCTGC
PstI-mNeoGreen-F	CTGCAGGGGAGCGGGAGCGGGAGCATGGTCAGCAAAGGTGAAGA
HindIII-mNeoGreen-R	AAGCTTCTACTTGTACAGTTCGTCCAT
KpnI-mNeoGreen-F	CCGACGAGAGGTACCATGGTCAGCAAAGGTGAAGA
BamHI-mNeoGreen-R	GTCGACTCTAGAGGATCCCTTGTACAGTTCGTCCAT
BamHI-QWRF1ProGUS-F	TGGCTGCAGGTCGACACAGACTAGAAAGGTATGCT
EcoRI-QWRF1ProGUS-R	GGTGGACTCCTCTTATATCTACGAGAAATGTCTCGC
BamHI-QWRF2ProGUS-F	TGGCTGCAGGTCGACCATTGTGTCTTTGAAACTAAA
EcoRI-QWRF2ProGUS-R	GGTGGACTCCTCTTATCTCGTCGGAAAAAACTATTC
QWRF1Gateway-F	TGGCTGCGATTCCTCAGGGGGGGG
QWRF1Gateway-R	ACCACTTTGTACAAGAAAGCTGGGTGTTATAGCCGGCTAAGTTGTATTATGTGC
QWRF2Gateway-F	GGGGACAAGTTTGTACAAAAAAGCAGGCTTCATGGTTGCTGCAGCGATTTC
QWRF1Gateway-R	GGGGACCACTTTGTACAAGAAAGCTGGGTGCTACAATTGCGGAGTCAAGCTGC
QWRF1-SP6	GATTTAGGTGACACTATAGAATGCTGGAGAAGCAACTGCCAAATC

QWRF1-T7	TGTAATACGACTCACTATAGGGGACCGTTTAGGTAACGAGGAAG
QWRF2-SP6	GATTTAGGTGACACTATAGAATGCTCTCTCCTCTCTCTCGTCTTCTT
QWRF2-T7	TGTAATACGACTCACTATAGGGCCGACATTTCAGTGCCTACA
